# Megakaryocytes participate in the occurrence of bleomycin-induced pulmonary fibrosis

**DOI:** 10.1038/s41419-019-1903-8

**Published:** 2019-09-09

**Authors:** Yan Zhou, Bo Zhang, Chen Li, XiaoTing Huang, HaiPeng Cheng, XingWen Bao, FeiYan Zhao, QingMei Cheng, ShaoJie Yue, JianZhong Han, ZiQiang Luo

**Affiliations:** 1 Department of Physiology, Xiangya School of Medicine, Central South University, Changsha, Hunan China; 2 Department of Urology, Xiangya Hospital, Central South University, Changsha, Hunan China; 3 Department of Physiology, Changzhi medical college, Changzhi, Shanxi China; 4 Department of Pediatrics, Xiangya Hospital, Central South University, Changsha, Hunan China

**Keywords:** Respiration, Respiratory tract diseases

## Abstract

Pulmonary fibrosis is characterized by the remodeling of fibrotic tissue and collagen deposition, which mainly results from aberrant fibroblasts proliferation and trans-differentiation to myofibroblasts. Patients with chronic myelogenous leukemia, myeloproliferative disorder, and scleroderma with pulmonary fibrosis complications show megakaryocyte infiltration in the lung. In this study, we demonstrated that the number of CD41^+^ megakaryocytes increased in bleomycin (BLM)-induced lung fibrosis tissues through the Chemokine (CXCmotif) ligand 12/Chemokine receptor 4 (CXCL12/CXCR4) axis. Pharmacological inhibition of the CXCL12/CXCR4 axis with WZ811 prevented migration of CD41^+^ megakaryocytes induced by BLM-injured lung tissue ex vivo and in vivo. In addition, WZ811 significantly attenuated lung fibrosis after BLM challenge. Moreover, megakaryocytes directly promoted fibroblast proliferation and trans-differentiation to myofibroblasts. We conclude that thrombopoietin (TPO) activated megakaryocytes through transforming growth factor β (TGF-β) pathway to promote fibroblast proliferation and trans-differentiation to myofibroblasts, which is abolished by treatment with selective TGF-βR-1/ALK5 inhibitors. Therefore, CD41^+^ megakaryocytes migrate to injured lung tissue partially through the CXCL12/CXCR4 axis to promote the proliferation and trans-differentiation of fibroblasts through direct contact and the TGF-β1 pathway.

## Introduction

Pulmonary fibrosis is a chronic, progressive, and irreversible lung disease characterized by the remodeling of fibrotic tissue and collagen deposition in the lung parenchyma, which ultimately leads to loss of lung function. Tissue fibroblasts and their active phenotype myofibroblast are generally thought to be responsible for repair and remodeling in the lung^1^. In recent years, the incidence and mortality of pulmonary fibrosis have been increasing, which is a serious threat to people’s health. The median survival time from diagnosis is only 2–4 years^2–4^. To date, the exact mechanism of pulmonary fibrosis progression remains unclear and there is still no effective treatment to reverse fibrosis^5^. Therefore, it is important to further unveil the underlying cellular and molecular mechanisms of pulmonary fibrosis for diagnosis and treatment.

Multifaceted evidence suggests that megakaryocyte residents in the bone marrow contribute to idiopathic myelofibrosis^6–10^. Reports demonstrated that megakaryocyte infiltration in cases of chronic myelogenous leukemia^11,12^ and myeloproliferative disorder^13,14^ is associated with the occurrence of pulmonary fibrosis. In addition, many studies have shown that megakaryocytes in the lungs are closely related to several respiratory diseases, including diffuse alveolar damage, burns-induced lung injury, and pulmonary fibrosis in scleroderma^15–18^. As early as 1893, Aschoff first observed the presence of megakaryocytes in the lungs^19^. One study provided evidence of all platelets produced by megakaryocytes in the pulmonary circulation^20^. Furthermore, a recent study reported that the lung may be a site of platelet production and a reservoir for hematopoietic progenitors^21^. However, the concrete role and mechanism of function of megakaryocytes in pulmonary fibrosis have not yet been elucidated.

In this study, we aimed to appraise the effect of megakaryocytes in BLM-induced pulmonary fibrosis and specifically evaluate its mechanism of participating in fibrosis progression and its effect on lung fibroblasts.

## Results

### CD41^+^ megakaryocyte counts increase in lung tissue of BLM-challenged mice

**Fig. 1:**
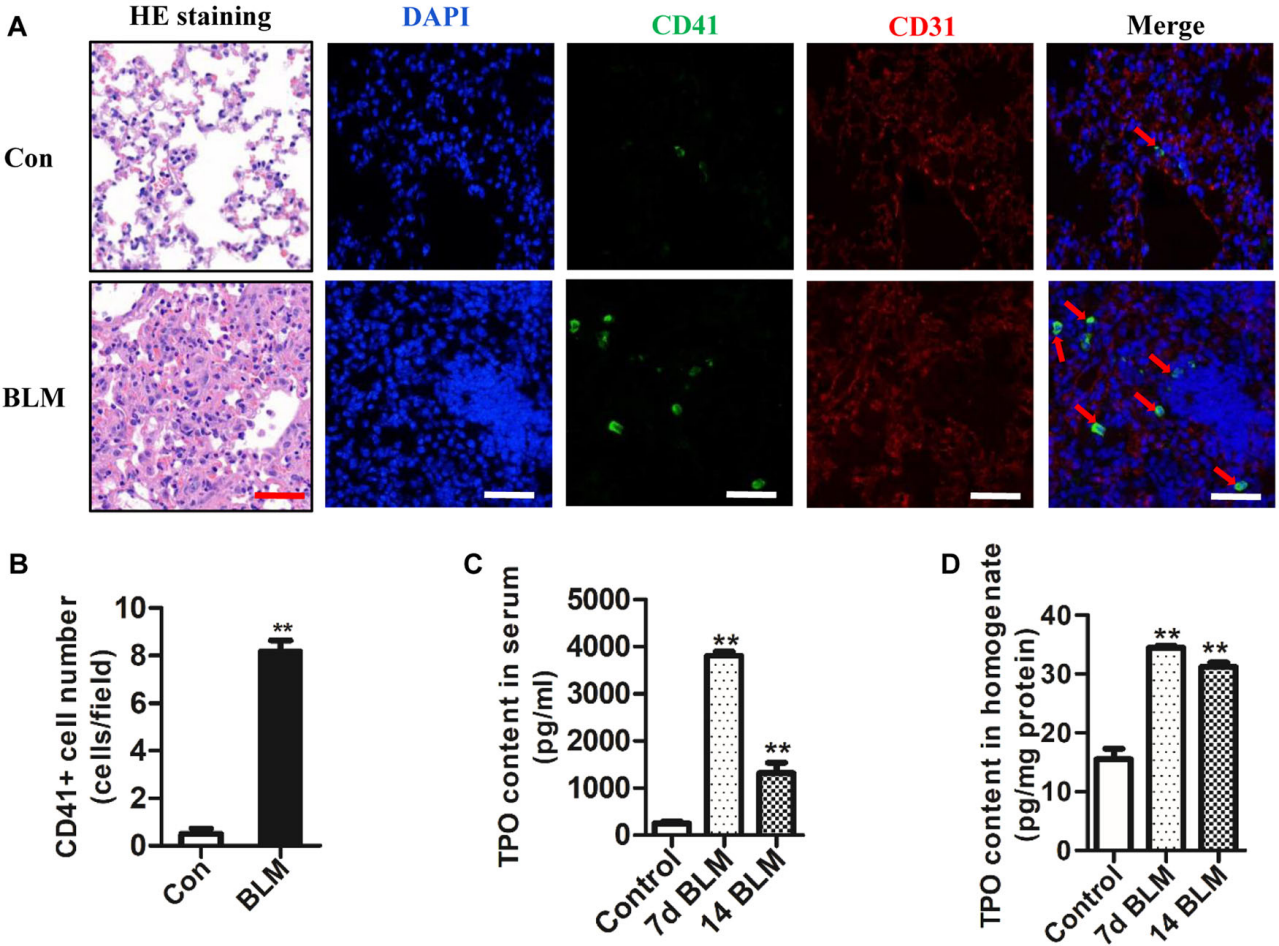
BLM increased the number of CD41^+^ megakaryocytes in lung tissue of mice. **a** The figure demonstrated a representative view of lung tissue on 14 days after the BLM injection (first panel: HE staining; second panel: DAPI; third panel: CD41 immunofluorescence; forth panel: CD31 immunofluorescence; fifth panel: Merge, × 400 magnification, CD41^+^ megakaryocytes marked by red arrow, Bar = 100 μm). **b** Data analysis of CD41^+^ megakaryocytes in lung tissue of mice (*n* = 3, **P < 0.01 vs control group). **c** The content of TPO protein in serum of mouse detected by ELISA (*n* = 3, **P < 0.01 vs control group). **d** The content of TPO protein in lung homogenate of mouse detected by ELISA (*n* = 3, **P < 0.01 vs control group). The data are presented as the mean ± SD

Histological examination using H&E staining of lung tissues revealed representative features of the fibrotic response with destruction of alveolar structure and interstitial thickening after BLM insult for 14 days (Fig. 1a). To investigate the number of megakaryocytes in the lung tissue of mice with BLM-induced lung fibrosis, CD41^+^ cells were measured by immunofluorescence. In order to distinguishing cells in intravascular and extravascular, we also signed the expressions of endothelial marker CD31 to mark the blood vessels. We observed that the number of CD41^+^ megakaryocytes increased in the lung tissue on day 14 after BLM challenge and almost increased megakaryocytes did not exist in blood vessels (Fig. 1a, b). In addition, thrombopoietin (TPO) is a crucial regulator of megakaryocytes maturation and activation, and our results also showed that the content of TPO significantly enhanced both in serum and in lung tissue after BLM insult for 7 day and 14 day compared with the control group (Fig. 1c, d). These data suggest that the increased in megakaryocytes and TPO level may be associated with the occurrence of BLM-induced fibrosis.

### BLM-injured lung tissue promotes megakaryocyte migration through CXCL12/CXCR4 axis

**Fig. 2:**
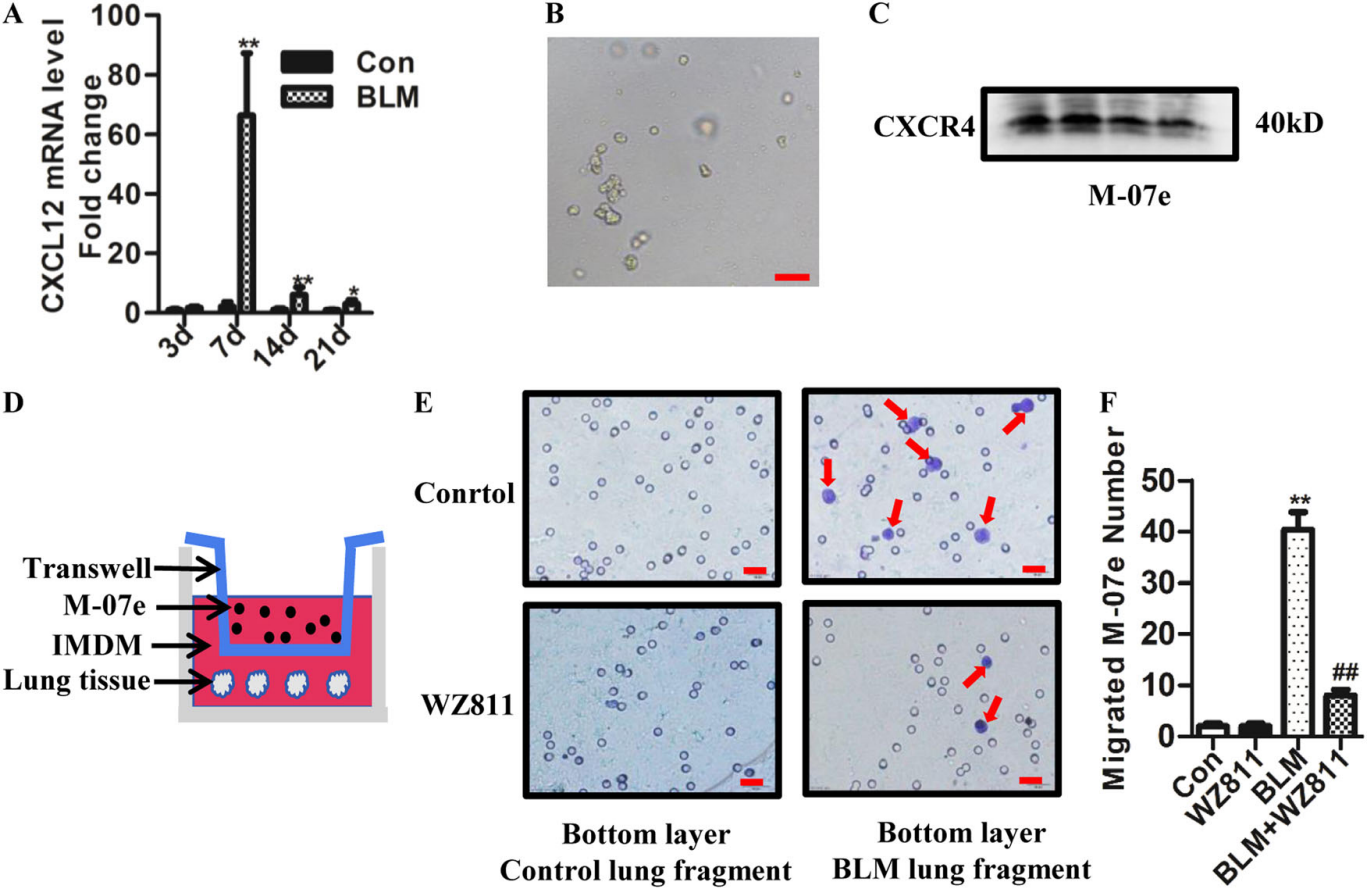
BLM-injured lung tissue promoted megakaryocytes migration through the CXCL12/CXCR4 axis. **a** The mRNA expression of CXCL12 in the lung tissue of mice was measured by qPCR (*n* = 4, *P < 0.05, **P < 0.01 vs control group). **b** Microscopic appearance of M-07e (Bar = 50 μm). **c** The expression of CXCR4 in M-07e was measured by western blotting. **d** Experimental design: transwell assay. **e** Representative images of migrated M-07e (crystal violet staining: purple, marked by red arrow, Bar = 20 μm). **f** Data analysis of migrated M-07e (*n* = 5, **P < 0.01 vs control group. ^##^*P* < 0.01 vs BLM group). The data are presented as the mean ± SD

Studies have demonstrated that the CXCL12/CXCR4 axis plays an essential role in the regulation of megakaryocyte (MK) migration^22–24^. To explore the mechanism responsible for the increased number of megakaryocytes in BLM-induced lung fibrosis, we measured CXCL12 expression in the lung tissue of mice after BLM challenge by quantitative real-time PCR (qPCR). Our results showed that compared with the control group, the expression of CXCL12 significantly increased in the lung tissue on 7, 14, and 21 days after BLM insult, which was significantly higher on 7 day after BLM challenge than other time point (Fig. 2a). In this study, we used M-07e, a megakaryocyte line, to explore how the megakaryocyte chemotaxis to injured lung tissues. Appearance of M-07e is shown in Fig. 2b. Our results showed that CXCR4, which is the receptor of CXCL12, exists in M-07e as determined by western blotting (Fig. 2c). To observe the migratory capacity of M-07e to injured lung tissues, we designed a transwell migration assay (Fig. 2d). When BLM-treated lung tissue on day 7 after BLM administration was incubated into the lower chamber, the quantified data showed that the migration number of M-07e significantly increased compared with normal lung tissue incubated in the lower chamber. However, administration with WZ811, a specific inhibitor of CXCL12/CXCR4 axis, significantly decreased the migration number of M-07e induced by BLM-treated lung tissues (Fig. 2e, f). These results suggest that megakaryocytes may migrate into the injured lung tissue through the CXCL12/CXCR4 axis.

### Pharmacological inhibition of CXCL12/CXCR4 axis attenuates BLM-induced pulmonary fibrosis partly through prevention of megakaryocyte migration

**Fig. 3:**
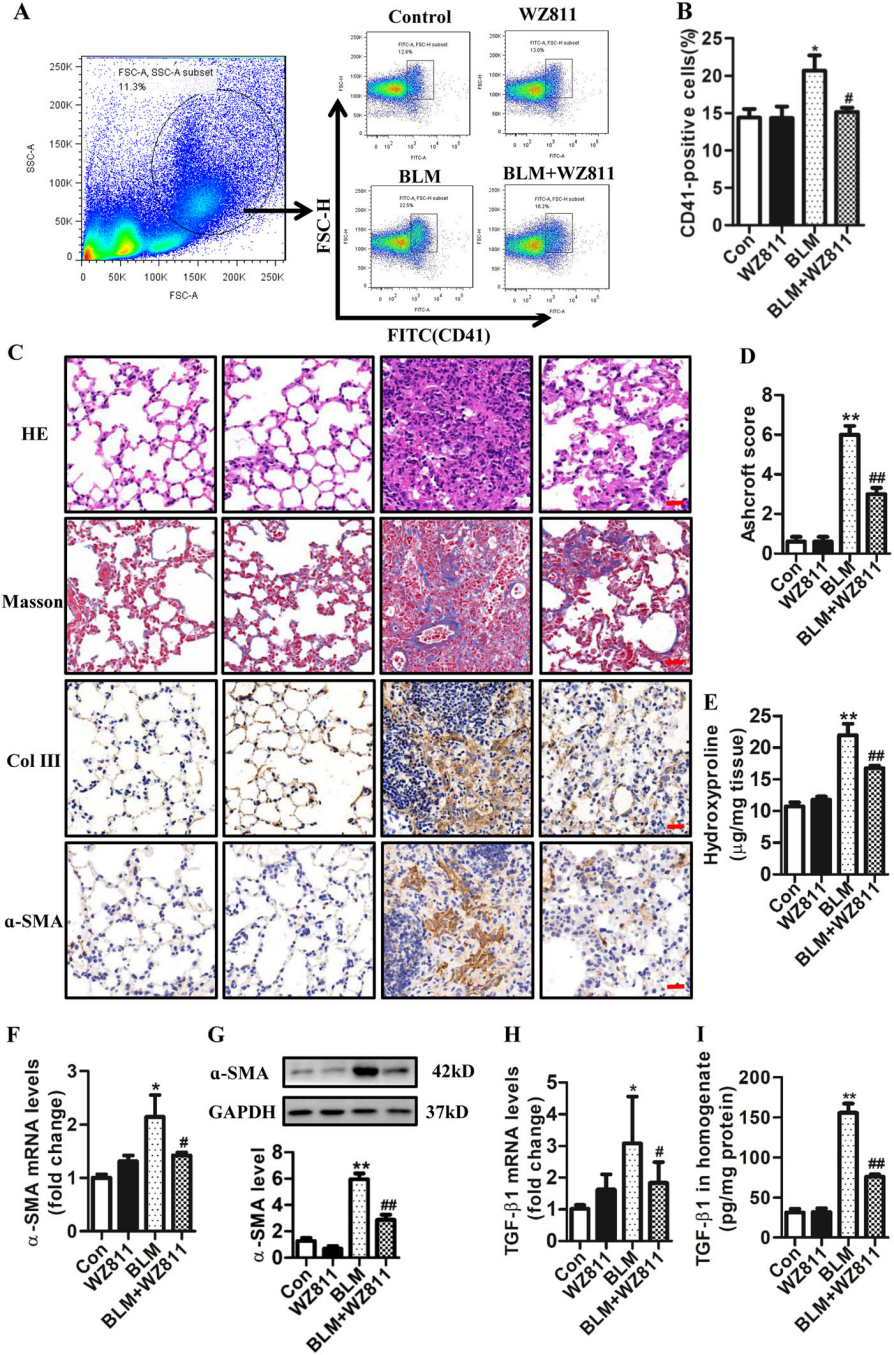
Effects of WZ811 on BLM-induced pulmonary fibrosis. **a** Flow cytometry analysis of CD41^+^ cells (megakaryocyte) in the lung tissues of different groups. **b** Relative percentage of CD41^+^ cells (megakaryocyte) in different groups (*n* = 3, *P < 0.05 vs control group. ^#^P < 0.05 vs BLM group). **c** Representative micrographs of H&E staining, Masson’s trichrome and immunohistochemical staining of lung sections for Col III and ɑ-SMA (Bar = 50 μm). **d** Ashcroft score was used to evaluate the degree of fibrosis (*n* = 5, **P < 0.01 vs control group. ^##^*P* < 0.01 vs BLM group). **e** Collagen content was measured by hydroxyproline (HYP) assay (*n* = 4, **P < 0.01 vs control group. ^##^P < 0.01 vs BLM group). **f** The expression of ɑ-SMA mRNA in the lung tissue of mice detected by qPCR (*n* = 3, *P < 0.05 vs control group. ^#^*P* < 0.05 vs BLM group). **g** The content of ɑ-SMA in lung tissue detected by western blotting (*n* = 3, **P < 0.01 vs control group. ^##^*P* < 0.01 vs BLM group). **h** The expression of TGF-β1 mRNA in the lung tissue of mice detected by qPCR (*n* = 3, *P < 0.05 vs control group. ^#^*P* < 0.05 vs BLM group). **i**The content of TGF-β1 protein in lung homogenate of mouse detected by ELISA (*n* = 3, **P < 0.01 vs control group. ^##^*P* < 0.01 vs BLM group). The data are presented as the mean ± SD

To further explore the effect of megakaryocytes in BLM-induced lung fibrosis, a CXCL12/CXCR4 inhibitor was used to treat BLM-challenged mice. Previous studies have reported that the CXCR4 antagonist prevented bleomycin-induced murine pulmonary fibrosis by inhibiting the recruitment and migration of bone marrow-derived fibrocytes to the injured lungs^25–27^. However, studies also demonstrated that the CXCL12/CXCR4 axis participated in the regulation of megakaryocyte migration^22–24^. Moreover, our previous results have showed that WZ811 can attenuate the migration of M-07e megakaryocytes induced by BLM-injured lung tissue ex vivo. To detect the effect of WZ811 on BLM-induced lung fibrosis in vivo, mice were intragastrical administered with WZ811 (4 mg/kg) for consecutive 14 days after BLM challenge. Our results showed that the number of CD41^+^ megakaryocytes was increased in BLM insulted mice. In contrast, WZ811 significantly attenuated the migration of CD41^+^ megakaryocytes into the injured lung (Fig. 3a, b). H&E staining showed that in the control and WZ811 groups, the lung structures were normal, which indicated that WZ811 (4 mg/kg/day) caused no obvious damage to the lung structure. In the BLM group, histological examination using H&E and Masson’s Trichrome staining of lung tissues revealed representative features of the BLM-induced fibrotic response with destruction of lung tissue structure and large amount of collagen deposition. Compared with those of the BLM model group, morphological alterations were significantly reduced by WZ811 administration (Fig. 3c, d). Moreover, the immunohistochemical staining results showed that the expression of collagen III, which is an important component of the extracellular matrix, markedly increased in lung tissues of the BLM group and was significantly reduced in the BLM + WZ811 group (Fig. 3c). We also detected the hydroxyproline (HYP) content to assess the level of collagen deposition in lung tissue of each group. Results showed that BLM induced an increase in the HYP content, which was significantly reduced by WZ811 (Fig. 3e). We also found that the level of ɑ-SMA (another important marker of lung fibrosis), as determined by immunohistochemical staining (Fig. 3c), qPCR (Fig. 3f), and western blotting (Fig. 3g) analyses, increased in the lung tissue of the BLM group compared to the control group. However, WZ811 treatment mitigated the mRNA (Fig. 3f) and protein (Fig. 3c, g) levels of ɑ-SMA induced by BLM. These results showed that blocking the migration of megakaryocyte with WZ811 through CXCL12/CXCR4 axis significantly attenuated fibrosis induced by BLM. In addition, TGF-β1, an important pro-fibrosis index was significantly increased on day 14 in the BLM group as compared with the control group (Fig. 3h, i). However, a significantly lower level of TGF-β in the lung tissue from the BLM + WZ811 group as compared with that of the BLM group was confirmed by qPCR (Fig. 3h) and enzyme-linked immunosorbent assay (ELISA; Fig. 3i).

### Megakaryocytes promotes the proliferation of primary lung fibroblasts and trans-differentiation to myofibroblasts

**Fig. 4:**
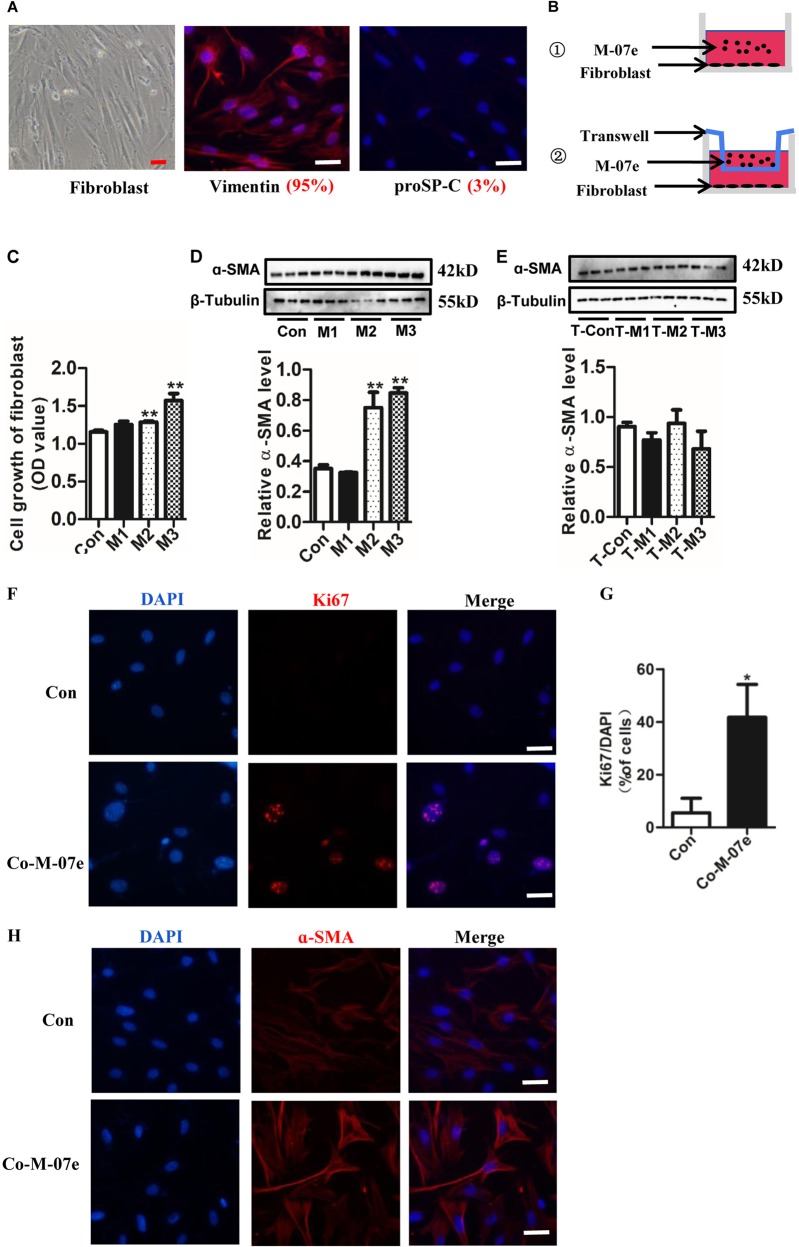
Effects of M-07e on the primary lung fibroblast. (**a**) Left: Microscopic appearance of primary lung fibroblasts (third passages); Middle: immunofluorescence for Vimentin; right: immunofluorescence for proSP-C (Bar = 30 μm). **b** Experimental design: ① direct co-culture assay; ② transwell co-culture assay. M-07e in the upper chamber, fibroblasts in the lower chamber. **c** Growth of primary lung fibroblasts, evaluated by the CCK-8 assay (*n* = 6, **P < 0.01 vs control group). Fibroblasts directly co-culture with different density of M-07e. M1: 5×10^3^/ml; M2: 10^4^/ml; M3: 2×10^4^/ml. **d** The level of ɑ-SMA evaluated by western blotting. Fibroblasts directly co-culture with different density of M-07e (*n* = 3, **P < 0.01 vs control group). M1: 5×10^3^/ml; M2: 10^4^/ml; M3: 2×10^4^/ml. **e** The level of ɑ-SMA evaluated by western blotting. Fibroblasts transwell co-culture with different density of M-07e (*n* = 3). M1: 5×10^3^/ml; M2: 10^4^/ml; M3: 2×10^4^/ml. **f** Immunofluorescence for Ki-67 of fibroblasts (Bar = 30 μm). Co-M-07e: 2×10^4^/ml M-07e co-culture with fibroblast. **g** Data analysis of the percentage of Ki-67^+^ to DAPI (*n* = 3, *P < 0.05 vs control group). **h** Immunofluorescence for ɑ-SMA of fibroblasts (Bar = 30 μm). The data are presented as the mean ± SD

Appearance and authentication of the isolated primary murine lung fibroblasts are shown in Fig. 4a. To further explore the effects of megakaryocytes, we designed three different experiments to detect the effect of megakaryocytes on fibroblasts. First, we performed fibroblasts direct co-culture with different density M-07e (Fig. 4b ①). Our results demonstrated that M-07e in the M2 and M3 groups enhanced cell growth of fibroblasts, as detected by the cell counting kit-8 (CCK-8) assay (Fig. 4c). In addition, M-07e in the M2 and M3 groups significantly increased the ɑ-SMA protein level as detected by western blotting, which is an important fibroblast marker for trans-differentiation to myofibroblasts (Fig. 4d). In addition, we used the transwell compartment to detect the indirect effects of megakaryocytes to lung fibroblasts (Fig. 4b ②). However, our results showed us that megakaryocytes had no obvious effects on the protein level of ɑ-SMA (Fig. 4e) in this indirect co-culture system. Furthermore, we performed immunofluorescence staining to detect Ki-67 (a proliferation marker) and ɑ-SMA expression. Our results showed that M-07e in the M3 group of the direct co-culture system (①) promoted expression of Ki-67 (Fig. 4f, g) and ɑ-SMA (Fig. 4h). These results indicated that M-07e directly promoted fibroblast proliferation and trans-differentiation to myofibroblasts. Furthermore, we isolated the primary fetal liver-derived megakaryocyte to revalidate these effects on fibroblast. Appearance of primary fetal liver-derived megakaryocyte is shown in Supplementary Fig. S1A. Our results showed that co-culture fibroblast with megakaryocyte enhanced cell growth of fibroblasts, as detected by the cell counting kit-8 (CCK-8) assay (Supplementary Fig. S1B). In addition, megakaryocyte significantly increased the ɑ-SMA protein level and Col III as detected by western blotting in fibroblasts (Supplementary Fig. S1C–E). We also performed immunofluorescence staining to detect the expression of ɑ-SMA in fibroblasts. Our results showed that primary megakaryocyte also promoted the expression of ɑ-SMA in fibroblasts (Supplementary Fig. S1F). We hypothesized that unstimulated megakaryocytes have direct contact effects on lung fibroblasts but cannot impact fibroblast trans-differentiation in an indirect way.

**Fig. 5:**
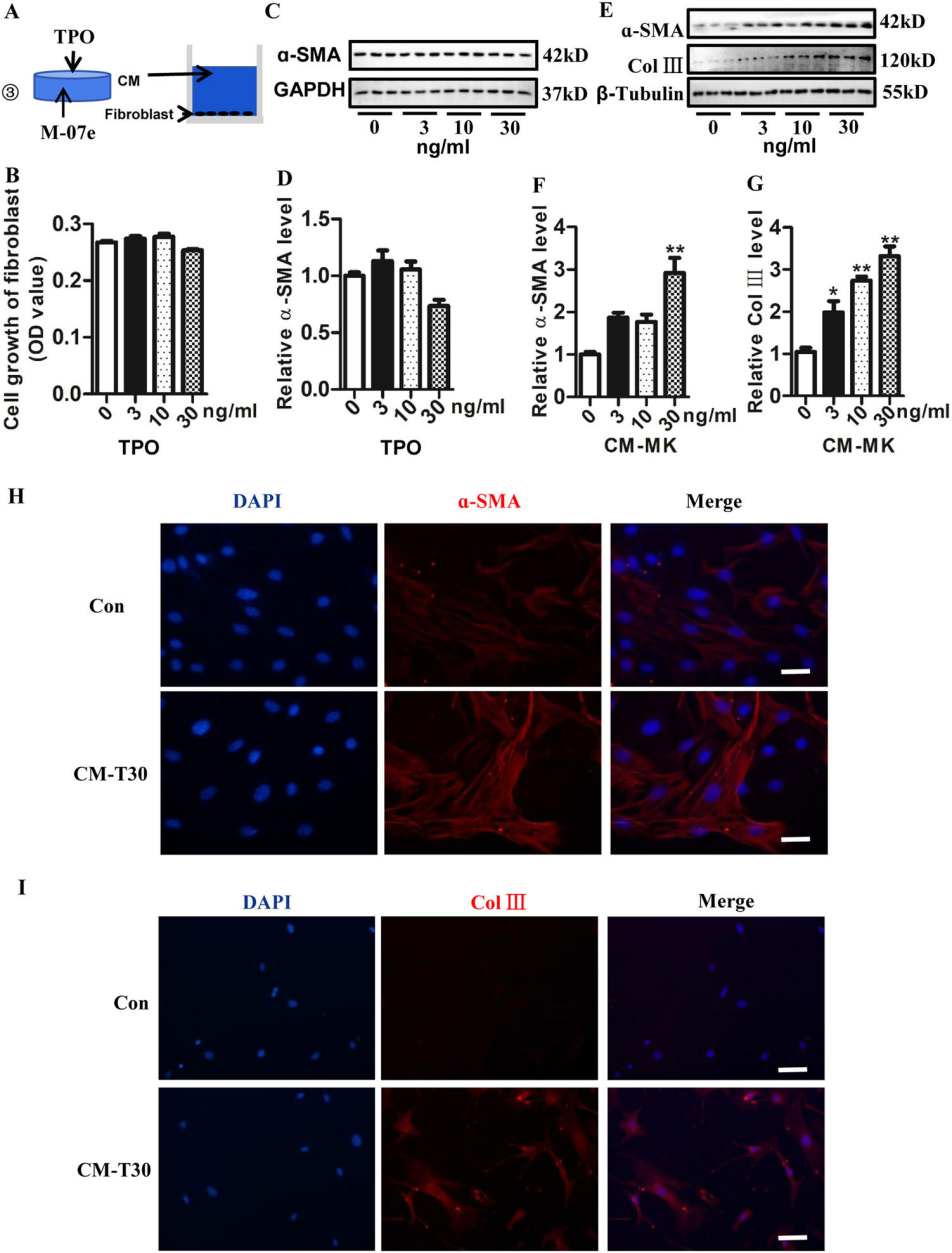
Effects of TPO-induced M-07e on the primary lung fibroblast. **a** Experimental design: ③ Conditioned medium (CM) of different concentration of TPO inducing M-07e to culture fibroblasts. **b** Cell growth of primary lung fibroblasts, evaluated by the CCK-8 assay (*n* = 6). Fibroblasts culture with different concentration TPO. **c** The level of ɑ-SMA evaluated by western blotting. Fibroblasts culture with different concentration TPO. **d** Semi-quantitative analysis of ɑ-SMA level evaluated by western blotting. Fibroblasts culture with different concentration TPO (*n* = 6). **e** The level of ɑ-SMA and Col III evaluated by western blotting. Fibroblasts culture with CM of M-07e inducing with different concentration TPO. **f** Semi-quantitative analysis of ɑ-SMA level evaluated by western blotting (*n* = 3, **P < 0.01 vs control group). **g** Semi-quantitative analysis of Col III level evaluated by western blotting (*n* = 3, *P < 0.05, **P < 0.01 vs control group). **h** Immunofluorescence for ɑ-SMA of fibroblasts cultured with CM of M-07e inducing with 30 ng/ml TPO (Bar = 30 μm, CM-T30 group: CM of 30 ng/ml TPO stimulation to M-07e). **i** Immunofluorescence for Col III of fibroblasts cultured with CM of M-07e inducing with 30 ng/ml TPO (Bar = 60 μm). The data are presented as the mean ± SD

Studies have shown that TPO as a major regulator of megakaryocyte maturation and activation in vivo^28^. To further detect the effects of TPO-stimulated active megakaryocytes on lung fibroblasts, different concentrations of TPO were used to stimulate and activate megakaryocytes, and then the fibroblasts were cultured with conditioned medium (CM) of M-07e (Fig. 5a). Our results showed that different concentration of TPO itself had no direct effect on fibroblast growth (Fig. 5b) and the protein level of ɑ-SMA in fibroblasts (Fig. 5c, d). However, our results showed that CM of 30 ng/ml TPO-stimulated M-07e significantly promoted the protein level of ɑ-SMA (Fig. 5e, f). Further, TPO-stimulated CM promoted the protein level of Col III in fibroblasts in a dose-dependent manner (Fig. 5e, g), as detected by western blotting. Moreover, our immunofluorescence staining results showed that CM of 30 ng/ml TPO-stimulated M-07e significantly promoted the protein level of ɑ-SMA (Fig. 5h) and Col III in fibroblasts (Fig. 5i).

### Megakaryocytes promotes proliferation of primary lung fibroblasts and trans-differentiation to myofibroblasts partially through TGF-β1 pathway

**Fig. 6:**
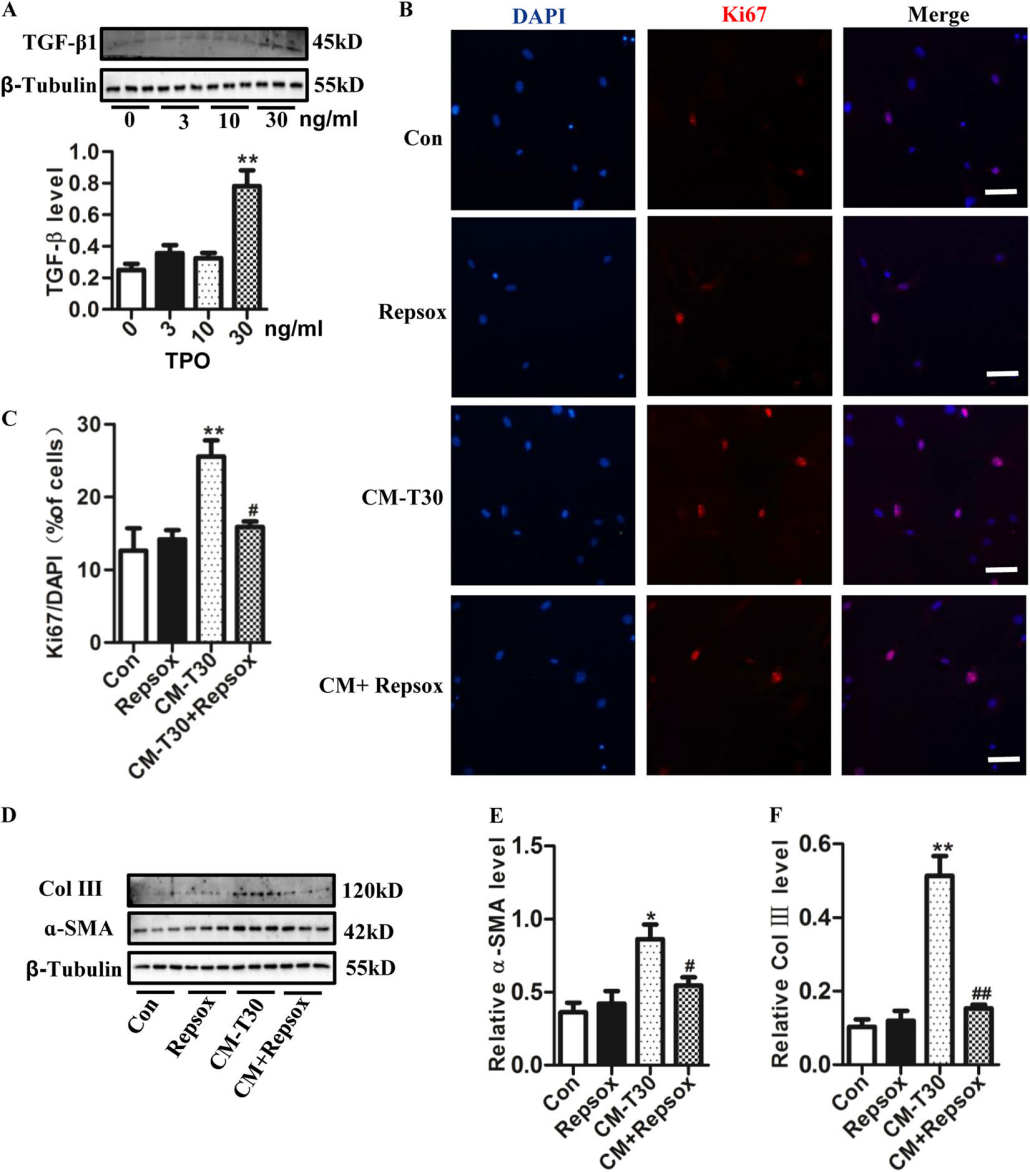
M-07e promoted the proliferation of primary lung fibroblast and trans-differentiation to myofibroblast through TGF-β1 pathway. **a** The level of TGF-β1 of M-07e inducing with different concentration TPO evaluated by western blotting (*n* = 3, **P < 0.01 vs control group). **b** Immunofluorescence for Ki-67 of fibroblasts cultured with CM of M-07e inducing with 30 ng/ml TPO with or not with Repsox (Bar = 60 μm). **c** Data analysis of the percentage of Ki-67^+^ to DAPI (*n* = 3, *P < 0.05, **P < 0.01 vs control group. #*P* < 0.05 vs CM-T30 group). **d** The level of ɑ-SMA and Col III evaluated by western blotting. **e** Semi-quantitative analysis of ɑ-SMA level evaluated by western blotting (*n* = 3, *P < 0.05 vs control group. ^#^*P* < 0.05 vs CM-T30 group). **f** Semi-quantitative analysis of Col III level evaluated by western blotting (*n* = 3, **P < 0.01 vs control group. ^##^*P* < 0.01 vs CM-T30 group). The data are presented as the mean ± SD

A role for TGF-β1 in the development of lung fibrosis has been widely proposed^1,29,30^. Megakaryocytes predominantly forced fibroblasts to produce extracellular matrix in the diseased conditions through production and secretion of several cytokines, such as transforming growth factor-β1 (TGF-β1), platelet-derived growth factor, or basic fibroblast growth factor^31^. TGF-β1 can be produced by cells within the megakaryocytic lineage in myelofibrosis patients and in macrophages^7^. To further detect the mechanism of the effect of megakaryocytes on lung fibroblasts, we detected the level of TGF-β1 in M-07e cells. Our results showed that the TGF-β1 level in M-07e significantly increased after 30 ng/ml TPO stimulation (Fig. 6a). Subsequently, we used Repsox, a specific inhibitor of TGF-β1, to further confirm whether megakaryocytes affected fibroblasts through the TGF-β1 pathway. Our results showed us that Repsox significantly attenuated the increasing expression of Ki-67 (Fig. 6b, c), ɑ-SMA (Fig. 6d, e) and Col III in fibroblast (Fig. 6d, f) induced by TPO-stimulated CM. Furthermore, we also detect the effect of CM of TPO-stimulated primary megakaryocytes on fibroblasts. Our results showed that CM of TPO-stimulated primary megakaryocytes significantly enhanced the expression of Ki-67 (Supplementary Fig. S2A, B) and ɑ-SMA in fibroblast (Supplementary Fig. S2C–E). However, Repsox significantly attenuated all these effects of CM of TPO-stimulated primary megakaryocytes. These results showed that TPO enabled TGF-β-expressing megakaryocytes to promote fibroblast proliferation and trans-differentiation, and Repsox, a specific inhibitor of TGF-β1 could block this effect.

## Discussion

Using a combination of BLM-induced lung fibrosis mouse models and primary lung fibroblasts obtained from newborn mice, we demonstrated that megakaryocytes participate in the occurrence of bleomycin-induced pulmonary fibrosis. We demonstrated that megakaryocytes promoted the occurrence of lung fibrosis. We observed that the number of CD41^+^ megakaryocytes and the level of TPO was markedly elevated in the lung tissue after BLM challenge, suggesting that the number of megakaryocytes increase in response to lung tissue injury. Our results showed that the injured lung tissue challenged by BLM expressed a higher level of CXCL12. We demonstrated that treatment with WZ811, a specific inhibitor of the CXCL12/CXCR4 axis, was sufficient to prevent migration of megakaryocytes induced by BLM-injured lung tissue ex vivo and in vivo. In addition, pharmacological inhibition of the CXCL12/CXCR4 axis with WZ811 significantly attenuated lung fibrosis after BLM challenge reflected by morphological changes, ɑ-SMA, Col III, and TGF-β1 levels, and HYP content. We then presented substantial evidence that megakaryocytes can directly promote fibroblast proliferation and trans-differentiation, while its mechanism needs to be further explored. However, TPO-stimulated active megakaryocyte can express TGF-β1 to promote fibroblast proliferation and trans-differentiation, which can be blocked by Repsox, a specific TGFβR-1/ALK5 inhibitor. These evidences indicate that megakaryocytes participate in lung fibrosis and promote fibroblast proliferation and trans-differentiation through direct contact and the TGF-β1 pathway. Our findings are significant to enhance our understanding of the pathogenesis of lung fibrosis and to encourage development of effective therapies.

It has previously been demonstrated that the pathogenesis of chronic myelogenous leukemia, myeloproliferative disorder and scleroderma with pulmonary fibrosis complications may be associated with megakaryocyte infiltration in the lung^11–14,18^. Our studies demonstrate that the number of CD41^+^ megakaryocytes increased in the lung tissue after BLM challenge, which is consistent with previously studies, suggesting that megakaryocytes may be associated with the occurrence of lung fibrosis.

CXCL12 is a member of the CXC chemokine family, which is a key chemokine in organisms. CXCR4 is the main receptor for CXCL12^32^. The CXCL12/CXCR4 axis plays an important role during embryogenesis in hematopoiesis, vascular development, cardiogenesis, and cerebellar development. Research has shown that the level of CXCL12 in BLM-induced lung fibrosis was significantly increased compared with the control group^33^. Clinical studies have shown that lung tissue and serum levels of CXCL12 are significantly elevated in patients with pulmonary fibrosis, and CXCL12 is detected in alveolar lavage fluid in 40% of patients with pulmonary fibrosis, whereas it is not detected in normal subjects^34^. Previous research has reported that CXCR4 antagonist AMD3100 prevented bleomycin-induced murine pulmonary fibrosis by inhibiting the fibrocyte mobilization to the injured lung^25–27^. Further, researches have also demonstrated that the CXCL12/CXCR4 axis participated in the regulation of megakaryocyte migration^22–24^. In our study, WZ811, another specific inhibitor, also attenuated BLM-induced lung fibrosis and megakaryocyte migration. Thus, our research suggests that pharmacological blocking of the CXCL12/CXCR4 axis can reduce megakaryocyte chemotaxis to damaged lung tissues, ultimately partially mitigating lung fibrosis.

Lung fibrosis is characterized by excess accumulation of the extracellular matrix (ECM), including collagens and fibronectin, resulting from an imbalance in ECM dynamics. This pathological accumulation of ECM is owing to the excess recruitment of fibroblasts to injured sites of lung tissues and to their excessive trans-differentiation to an effector myofibroblast phenotype^1^. Obviously, aberrant proliferation of fibroblasts and their trans-differentiation to myofibroblasts lead to progressive lung fibrosis, which is characterized by an excessive ECM build-up, leading to distortion of tissue architecture and ultimately lung failure^2–4^. It has been shown that megakaryocytes contributed to the bone marrow-matrix environment by expressing fibronectin, type IV collagen, and laminin^35^. Research has shown that TPO is a major regulator of megakaryocyte maturation and activation in vivo^28^. Megakaryocytes predominantly forced fibroblasts to produce ECMs in disease, through production and secretion of several cytokines, such as TGF-β1, platelet-derived growth factor, or basic fibroblast growth factor^31^. Our results suggest that megakaryocyte can direct promote fibroblast proliferation as detected by CCK-8 and Ki-67 expression and trans-differentiation to myofibroblasts indicated by the elevated expression of ɑ-SMA and Col III, while its mechanism needs to be further explored. In addition, we also demonstrate that TPO can active megakaryocyte and promote TGF-β1 expression. The CM of TPO-activated megakaryocytes promotes fibroblast proliferation and trans-differentiation to myofibroblasts, which are blocked by Repsox. Consequently, our results suggest that megakaryocytes directly or partially through the TGF-β1 pathway promote fibroblast proliferation and trans-differentiation to myofibroblasts.

**Fig. 7:**
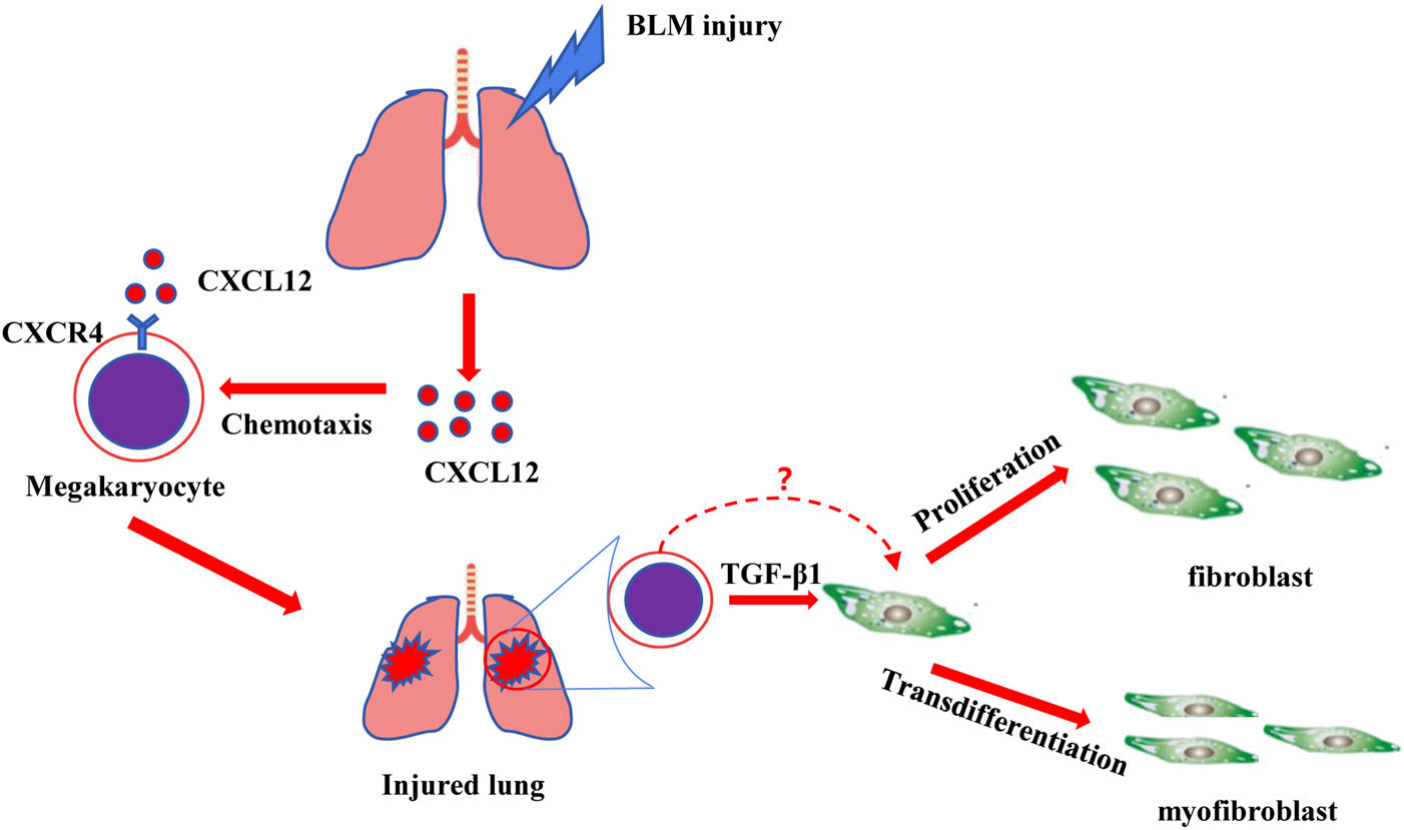
Schematic of a model of megakaryocyte involvement in pathophysiological processes of pulmonary fibrosis

In summary, we provide a new mechanistic insight into lung fibrosis and determine that megakaryocytes migrate to the BLM-injured lung through the CXCL12/CXCR4 axis and megakaryocytes promote the proliferation and trans-differentiation of fibroblasts partially through direct contact or TGF-β1 pathway (Fig. 7). However, as the pleiotropic effects of CXCR4 inhibition, using of the specific blockers of megakaryocyte recruitment in future studies is warranted.

## Materials and methods

### Ethics statement

The ethics committee of the Central South University Science Research Center (Changsha, China) approved the experiments in this study, which were conducted in accordance with the guidelines of the National Institutes of Health. Mice were anesthetized with sodium pentobarbital (80 mg kg^−1^, intraperitoneal injection) and every effort was made to minimize suffering before proceeding.

### Experimental animals and treatment

Animal experiments were conducted on 8-week-old male C57BL/6 mice (specific-pathogen-free [SPF] grade; Department of laboratory animal unit of Central South University, Changsha, China). The mice were intratracheally injected with 50 μL BLM (Nippon Kayaku, Tokyo, Japan) at 1.25 mg kg^−1^ dissolved with sterile phosphate buffer saline (PBS) or sterile PBS as control after being anesthetized. The mice in the control and BLM groups were killed on Days 3, 7, 14, and 21 after BLM administration, operating cardiac perfusion to clear blood from the lungs before removing the lung tissue.

To study the antifibrotic effects of WZ811 (Selleck, USA), which is a specific CXCR4 antagonist, mice were randomly assigned to four groups: (1) control group, (2) WZ811 group, (3) BLM group, and (4) BLM + WZ811 group. Mice in the WZ811 group and BLM + WZ811 group were intragastric administration with WZ811 (i.g., 4 mg/kg) for 14 consecutive days after BLM challenge. The mice in the control and BLM groups were intragastric administration with equal volumes of 0.9% NaCl solution. Lung samples were collected on Day 14 after BLM administration for further experiments or stored at −80 °C until further analysis.

### Histopathology evaluation

The upper right lobe of the lungs was isolated and fixed with 4% paraformaldehyde solution and then embedded in paraffin for the preparation of tissue sections for pathological examination. The sections were examined after being stained with hematoxylin and eosin (H&E) or Masson’s trichrome. Morphological changes in fibrotic lungs were quantified according to the criteria proposed by Ashcroft^36^. Grading was scored on a scale from 0 to 8, using the average of microscope field scores.

### Hydroxyproline assay

The collagen content in whole lungs was examined by measuring hydroxyproline (HYP) content. HYP content was measured using HYP kits (Jiancheng Biotechnology Institute, Nanjing, China) according to the manufacturer’s protocol.

### Quantitative real-time PCR

Total RNA was extracted from lung tissues with TRIzol reagent (Takara, Japan) according the manufacturer’s protocol. Total RNA (1 μg) was reverse transcribed into cDNA using a First Strand cDNA Synthesis Kit (Takara, Japan) following the manufacturer’s instructions. SYBR Green signals were detected using a Bio-Rad real-time PCR system (CFX96 Touch™, Bio-Rad, USA). The relative expression of mRNA was determined by normalizing the expression of each gene to Glyceraldehyde-3-phosphate dehydrogenase (*GAPDH*) gene following the 2–^ΔΔ^Ct method. The primer sequences are shown in Table 1.

**Table 1:** Sequences of specific primers (mouse) used in this study

Gene	Forward primer sequence (5 to 3)	Reverse primer sequence (5 to 3)	Length (bp)
GAPDH	gaaggtggtgaagcaggcatct	cggcatcgaaggtggaagagtg	116
ɑ-SMA	cttcgctggtgatgatgctc	gttggtgatgatgccgtgtt	175
TGF-β1	ttgcttcagctccacagaga	tggttgtagagggcaaggac	183

### Western blot analysis

Total protein lysates were extracted from the lung tissues of mice and cells using RIPA lysis buffer (Beyotime Biotechnology, China) plus proteinase inhibitor cocktail (Roche Diagnostics, Indianapolis, IN). The total protein concentration was measured using a BCA kit (Thermo Scientific, USA). The total proteins were separated using SDS-PAGE and electrotransferred to PVDF membranes (Millipore, USA). After blocking in 5% fat-free milk for 2 h, the membranes were incubated with the primary anti-β-tubulin (1:1000; Servicebio, China) or anti-GAPDH (1:2000; Servicebio, China) or anti-ɑ-smooth muscle actin (ɑ-SMA) (1:500; Servicebio, China) or anti-collagen III (Col III) (1:500; Servicebio, China) or anti-TGF-β1 (1:500; Cusabio, China) or anti-CXCR4 (1:500; Cusabio, China) antibodies overnight at 4 °C. The corresponding horseradish-peroxidase (HRP) secondary antibody (1:7500; Abcam, USA) was applied for 1 h at room temperature. Immunoreactive bands were detected with enhanced chemiluminescence reagents (Millipore) in the Molecular Imager ChemiDoc XRS System (Bio-Rad, USA). The abundance of targeted protein was analyzed using Image Lab analysis software by normalizing the protein level of β-tubulin or GAPDH. All experiments were performed at least in triplicate.

### Immunohistochemistry

Briefly, paraffin sections (thickness, 5 μm) were deparaffinized, rehydrated, immersed in 3% hydrogen peroxide for 10 min, and then incubated for 30 min in blocking buffer (5% bovine serum albumin). The sections were incubated overnight at 4 °C with antibodies against ɑ-SMA (1:100; Servicebio, China) or Collagen III (1:100; Servicebio, China), followed by incubation at room temperature with a horseradish-peroxidase (HRP)-conjugated secondary antibody (1:100; Sigma-Aldrich, USA). Images were acquired by a microscope (Nikon, Japan).

### Immunofluorescence staining

The lung tissue sections were deparaffinized in a xylene series and rehydrated through a decreasing ethanol series for immunofluorescence staining. The slides were pretreated by microwave in citrate buffer (100 mM, pH 7.0) for 10 min and washed 3 times with PBS. In brief, 3% H_2_O_2_ was used to eliminate endogenous peroxidase activity. For immunofluorescence staining of fibroblasts, cells were grown on coverslips and cultured under different conditions. Cells were washed twice with PBS, fixed with 4% paraformaldehyde for 15 min and then washed twice with PBS.

Slides and cells were blocked with 5% bovine serum albumin for 30 min. They were then incubated overnight at 4 °C in anti-CD41 (1:50, R&D, USA), anti-CD31 (1:100; Servicebio, China), anti-Vimentin (1:100; Servicebio, China), anti-proSP-C (1:100; Servicebio, China), anti-Ki-67 (1:100; Servicebio, China), anti-ɑ-SMA (1:100; Servicebio, China), or anti-Collagen III (1:100; Servicebio, China) antibody, followed by staining with corresponding secondary, antibodies (1:200, Beyotime, China) at room temperature. 4′,6-diamidino-2-phenylindole (DAPI; Proteintech, China) was used to dye cell nucleus. Images were acquired by a fluorescence microscope (Nikon, Japan). Analysis of at least five fields for the cells staining positive for Vimentin, proSP-C and Ki-67 were counted as a percentage of the total cell number (DAPI positive). Analysis of at least five fields for the cells staining positive for CD41 were counted per field, and using the average of microscope field number for analysis.

### Flow cytometry

The method to prepare the single-cell suspension of whole lung is as described previously^37^. Megakaryocytes were labeled with FITC anti-mouse CD41 (BD Biosciences, USA) at 4 °C for 20 min in 100 μl PBS. Cell staining was analyzed using a FACSCanto II (Becton Dickinson, USA). Data were analyzed using FlowJo software (FlowJo, USA).

### Enzyme-linked immunosorbent assay

ELISA was used to determine the TGF-β1 levels in the lung homogenate and thrombopoietin (TPO) levels in the lung homogenate and in serum. After thoracotomy, the lungs were removed and homogenized in PBS containing protease inhibitors (Thermo Fisher Scientific, USA). The lung homogenates were centrifuged at 10,000 × *g* to remove insoluble debris. The supernatants of lung homogenates were assayed with anti-mouse TGF-β1 ELISA kits (MULTISCIENCES(LIANKE) BIOTECH, CO., LTD, China) in accordance with the manufacturers’ instructions. The supernatants of lung homogenates and serum were assayed with anti-mouse TPO ELISA kits (Elabscience Biotechnology Co., Ltd, China) according to the manufacturer’s instructions.

### M-07e culture

M-07e is a human megakaryocyte cell line, purchased from Cellcook Biotech (Guangzhou, China), which is a suspension cell. These cells were cultured in Iscove’s Modified Dulbecco’s Medium (IMDM) (Procell,China) supplemented with 10% fetal calf serum (FBS) (CellMax, Australia) and 10 ng/ml Granulocyte-macrophage colony stimulating factor (GM-CSF) (Peprotech,USA) in a humidified 5% CO_2_ incubator at 37 °C.

### Transwell migration assay

The migration of M-07e was conducted in a transwell system. The lower surfaces of the filter in 8.0 μm pore size transwell inserts with 6.5 mm diameter (Corning, USA). We seeded 1 × 10^4^ M-07e in 200 μl serum-free IMDM with 10 ng/ml GM-CSF onto the upper transwell compartments in 24-well plates. The normal or BLM-induced lung tissue on day 7 after BLM administration was harvested, cut into pieces (1 mm^3^), and incubated into the lower chamber, with/without WZ811 (1 μmol/L, Selleck, USA) added to the upper chambers to treat M-07e. The cells were allowed to migrate for 12 h. After washing and removing the remaining M-07e on the upper surface of the filter with a swab, the migrated M-07e were observed and counted after 0.1% crystal violet staining (Solarbio, China) and photographed with a microscope (Nikon, Japan).

### Fibroblast isolation and culture

Newborn 3–7 days C57BL/6 mice were killed, and the lungs from the thoraxes were obtained under sterile conditions. The lung was washed twice with sterile PBS containing 20% concentration of penicillin streptomycin mixing liquid (Solarbio, China). Subsequently, the lung tissue was chopped with surgical scissors until finely minced, digested with 2 ml enzyme mix (1 mg/ml Collagenase I and 10 μg/ml DNase I (Solarbio, China), and incubated at 37 °C for 1 h with gentle agitation. Further, an 80 μm cell strainer was used to filter the cells. Following, the cells were resuspended in Dulbecco’s modified Eagle’s media and Ham’s F-12 (DMEM/F-12) (Hyclone, USA) containing 10% fetal calf serum (FBS) (CellMax, Australia), 1% penicillin streptomycin mixing liquid (Solarbio, China) and incubated at 37 °C in a humidified atmosphere of 5% CO_2_. We performed anti-Vimentin immunofluorescence staining to identify cells for >90% fibroblasts, and performed anti-Prosurfactant protein C (proSP-C) immunofluorescence staining to identify cells for <5% alveolar epithelial cells. Cells were used between the third and sixth passages and photographed.

### Primary fetal liver-derived megakaryocytes isolation and culture

Mouse fetal liver on embryonic day 14 (E14) was collected according to the protocol^38^, passing through 18-gauge and 22-gauge needles, followed by filtering with a 40-μm cell strainer. According to the protocol, we use 10 ml DMEM containing 10% fetal calf serum (FBS) (CellMax, Australia), 1% penicillin streptomycin mixing liquid (Solarbio, China) to culture the single-cell suspension cells in 37 °C incubator for 4 days. Then use a triple gradient of bovine serum albumin (BSA; 4%, 3%, and 1.5%) for first-pass enrichment for MKs were performed as described previously^39^. Purified MKs was photographed and observed, and used to co-culture with fibroblasts.

### Co-culture megakaryocytes with fibroblasts

We designed three experiments to detect the effect of megakaryocytes on Fibroblasts: ① Culture Fibroblasts with different density M-07e (M1:5 × 10^3^/ml; M2: 10^4^/ml; M3: 2 × 10^4^/ml) in IMDM with 10 ng/ml GM-CSF containing 2% fetal calf serum (FBS) for 24 h for CCK8 assay or 36 h for protein level detection (western blotting or immunofluorescence staining); ② Culture Fibroblasts with different density M-07e (M1: 5 × 10^3^/ml; M2: 10^4^/ml; M3: 2 × 10^4^/ml) in IMDM with 10 ng/ml GM-CSF containing 2% fetal calf serum (FBS) in a transwell compartment for 36 h. Upper chamber: different density M-07e; Lower chamber: Fibroblasts; ③ Stimulated megakaryocytes in IMDM with 10 ng/ml GM-CSF containing 2% fetal calf serum (FBS) with different concentration of TPO (0, 3, 10, and 30 ng/ml, Peprotech, USA) for 24 h and cultured fibroblasts with conditioned medium (CM) of different concentration of TPO stimulation to M-07e for 36 h. To detect the effects of Repsox (1 μmol/L, Selleck, USA), which is a selective TGF-βR-1/ALK5 inhibitor, fibroblasts were randomly assigned to four groups: (1) Control group, (2) Repsox, (3) CM-T30 group (CM of 30 ng/ml TPO stimulation to M-07e), (4) CM + Repsox group.

Furthermore, we use primary fetal liver-derived murine megakaryocytes (density: 10^4^/ml) to direct co-culture (①) with fibroblasts for evaluating the effect of primary megakaryocytes on fibroblast. In addition, stimulated megakaryocytes in DMEM with TPO (30 ng/ml, Peprotech, USA) for 24 h and cultured fibroblasts with conditioned medium (CM) of TPO stimulation to primary megakaryocytes for 48 h(③) with or without Repsox (1 μmol/L, Selleck, USA).

### Cell growth assay

Cell growth was measured by the Cell Counting Kit-8 (CCK-8) assay, which is based on the conversion of an orange-colored product from water-soluble tetrazolium salt (WST-8) by dehydrogenases in live cells. For various treatment conditions, primary lung fibroblasts were plated in 96-well plates. Primary lung fibroblasts growth was investigated using a Cell Counting Kit 8 (YeSen, China) according to the manufacturer’s instructions.

### Statistical analysis

Statistical comparisons between two groups were analyzed with unpaired Student’s *t*-test. Comparisons among multiple groups were assessed with one-way ANOVA, followed by Student–Newman–Keuls (SNK) test for multiple comparisons. The data have been presented as mean ± SD. *P* values < 0.05 were statistically significant.

## Supplementary information

Supplementary information.

## References

[1] Lagares D (2017). ADAM10-mediated ephrin-B2 shedding promotes myofibroblast activation and organ fibrosis. Nat. Med..

[2] Richeldi L, Collard HR, Jones MG (2017). Idiopathic pulmonary fibrosis. Lancet.

[3] Lederer DJ, Martinez FJ (2018). Idiopathic pulmonary fibrosis. N. Engl. J. Med..

[4] Kropski JA, Blackwell TS (2019). Progress in understanding and treating idiopathic pulmonary fibrosis. Annu. Rev. Med..

[5] Mora AL, Rojas M, Pardo A, Selman M (2017). Emerging therapies for idiopathic pulmonary fibrosis, a progressive age-related disease. Nat. Rev. Drug Discov..

[6] Wen QJ (2015). Targeting megakaryocytic-induced fibrosis in myeloproliferative neoplasms by AURKA inhibition. Nat. Med..

[7] Ciurea SO (2007). Pivotal contributions of megakaryocytes to the biology of idiopathic myelofibrosis. Blood.

[8] Villeval JL (1997). High thrombopoietin production by hematopoietic cells induces a fatal myeloproliferative syndrome in mice. Blood.

[9] Shivdasani RA, Fujiwara Y, McDevitt MA, Orkin SH (1997). A lineage-selective knockout establishes the critical role of transcription factor GATA-1 in megakaryocyte growth and platelet development. EMBO J..

[10] Vannucchi AM (2002). Development of myelofibrosis in mice genetically impaired for GATA-1 expression (GATA-1(low) mice). Blood.

[11] Yamauchi K, Shimamura K (1994). Pulmonary fibrosis with megakaryocytoid cell infiltration and chronic myelogenous leukemia. Leuk. Lymphoma.

[12] Yamauchi K, Oda K, Shimamura K, Arimori S, Nagao T (1993). Pulmonary fibrosis with megakaryocytoid cell infiltration in accelerated phase of chronic myelogenous leukaemia. Br. J. Haematol..

[13] Rosenstingl S, Brouland JP, Zini JM, Tobelem G, Dupuy E (2003). Mixed myelodysplastic syndrome and myeloproliferative disorder with bone marrow and pulmonary fibrosis: the role of megakaryocytes. Acta Haematol..

[14] Yamauchi K, Shimamura K (1996). Pulmonary fibrosis with megakaryocyte infiltration in agnogenic myeloid metaplasia with thrombocytosis. Eur. J. Haematol..

[15] Martin JF, Slater DN, Trowbridge EA (1983). Abnormal intrapulmonary platelet production: a possible cause of vascular and lung disease. Lancet.

[16] Mandal RV, Mark EJ, Kradin RL (2007). Megakaryocytes and platelet homeostasis in diffuse alveolar damage. Exp. Mol. Pathol..

[17] Wells S, Sissons M, Hasleton PS (1984). Quantitation of pulmonary megakaryocytes and fibrin thrombi in patients dying from burns. Histopathology.

[18] Thachil J (2009). The lung megakaryocytes and pulmonary fibrosis in systemic sclerosis. Med. Hypotheses.

[19] Zhou Y, Huang YH, Luo ZQ (2017). [The platelet-producing function of lung]. Sheng Li Xue Bao.

[20] Trowbridge EA, Martin JF, Slater DN (1982). Evidence for a theory of physical fragmentation of megakaryocytes, implying that all platelets are produced in the pulmonary circulation. Thromb. Res..

[21] Lefrancais E (2017). The lung is a site of platelet biogenesis and a reservoir for haematopoietic progenitors. Nature.

[22] Mazharian A (2012). Assessment of megakaryocyte migration and chemotaxis. Methods Mol. Biol..

[23] Feng Y (2012). Umbilical cord blood-derived stromal cells regulate megakaryocytic proliferation and migration through SDF-1/PECAM-1 pathway. Cell Biochem. Biophys..

[24] Salim JP (2017). Differential expression of SDF-1 receptor CXCR4 in molecularly defined forms of inherited thrombocytopenias. Platelets.

[25] Song JS (2010). Inhibitory effect of CXC chemokine receptor 4 antagonist AMD3100 on bleomycin induced murine pulmonary fibrosis. Exp. Mol. Med..

[26] Makino H (2013). Antifibrotic effects of CXCR4 antagonist in bleomycin-induced pulmonary fibrosis in mice. J. Med. Invest..

[27] Chow LN (2016). Impact of a CXCL12/CXCR4 Antagonist in Bleomycin (BLM) Induced Pulmonary Fibrosis and Carbon Tetrachloride (CCl4) Induced Hepatic Fibrosis in Mice. PLoS ONE.

[28] Malara A (2019). EDA fibronectin-TLR4 axis sustains megakaryocyte expansion and inflammation in bone marrow fibrosis. J. Exp. Med..

[29] Wei Y (2017). Fibroblast-specific inhibition of TGF-beta1 signaling attenuates lung and tumor fibrosis. J. Clin. Invest..

[30] Palumbo-Zerr K (2015). Orphan nuclear receptor NR4A1 regulates transforming growth factor-beta signaling and fibrosis. Nat. Med..

[31] Malara A (2015). The secret life of a megakaryocyte: emerging roles in bone marrow homeostasis control. Cell Mol. Life Sci..

[32] Zhao F (2019). Pretreatment with G-CSF Could Enhance the Antifibrotic Effect of BM-MSCs on Pulmonary Fibrosis. Stem Cells Int..

[33] Hashimoto N, Jin H, Liu T, Chensue SW, Phan SH (2004). Bone marrow-derived progenitor cells in pulmonary fibrosis. J. Clin. Invest..

[34] Mehrad B (2007). Circulating peripheral blood fibrocytes in human fibrotic interstitial lung disease. Biochem. Biophys. Res. Commun..

[35] Malara A (2014). Megakaryocytes contribute to the bone marrow-matrix environment by expressing fibronectin, type IV collagen, and laminin. Stem Cells.

[36] Ashcroft T, Simpson JM, Timbrell V (1988). Simple method of estimating severity of pulmonary fibrosis on a numerical scale. J. Clin. Pathol..

[37] Zhou Y (2018). Omentin-1 protects against bleomycin-induced acute lung injury. Mol. Immunol..

[38] Vijey P, Posorske B, Machlus KR (2018). In vitro culture of murine megakaryocytes from fetal liver-derived hematopoietic stem cells. Platelets.

[39] Chen Z, Hu M, Shivdasani RA (2007). Expression analysis of primary mouse megakaryocyte differentiation and its application in identifying stage-specific molecular markers and a novel transcriptional target of NF-E2. Blood.

