# Supplementary material for: Megakaryocytes participate in the occurrence of bleomycin-induced pulmonary fibrosis
Source: Cell Death Dis. 2019 Sep 9;10(9):648. doi: 10.1038/s41419-019-1903-8 (PMC6733875; doi:10.1038/s41419-019-1903-8)
Supplement: Supplementary file 1 — Supplementary information. [file 41419_2019_1903_MOESM1_ESM.docx]

**Supplementary information**

**Supplementary Figure S1**

**
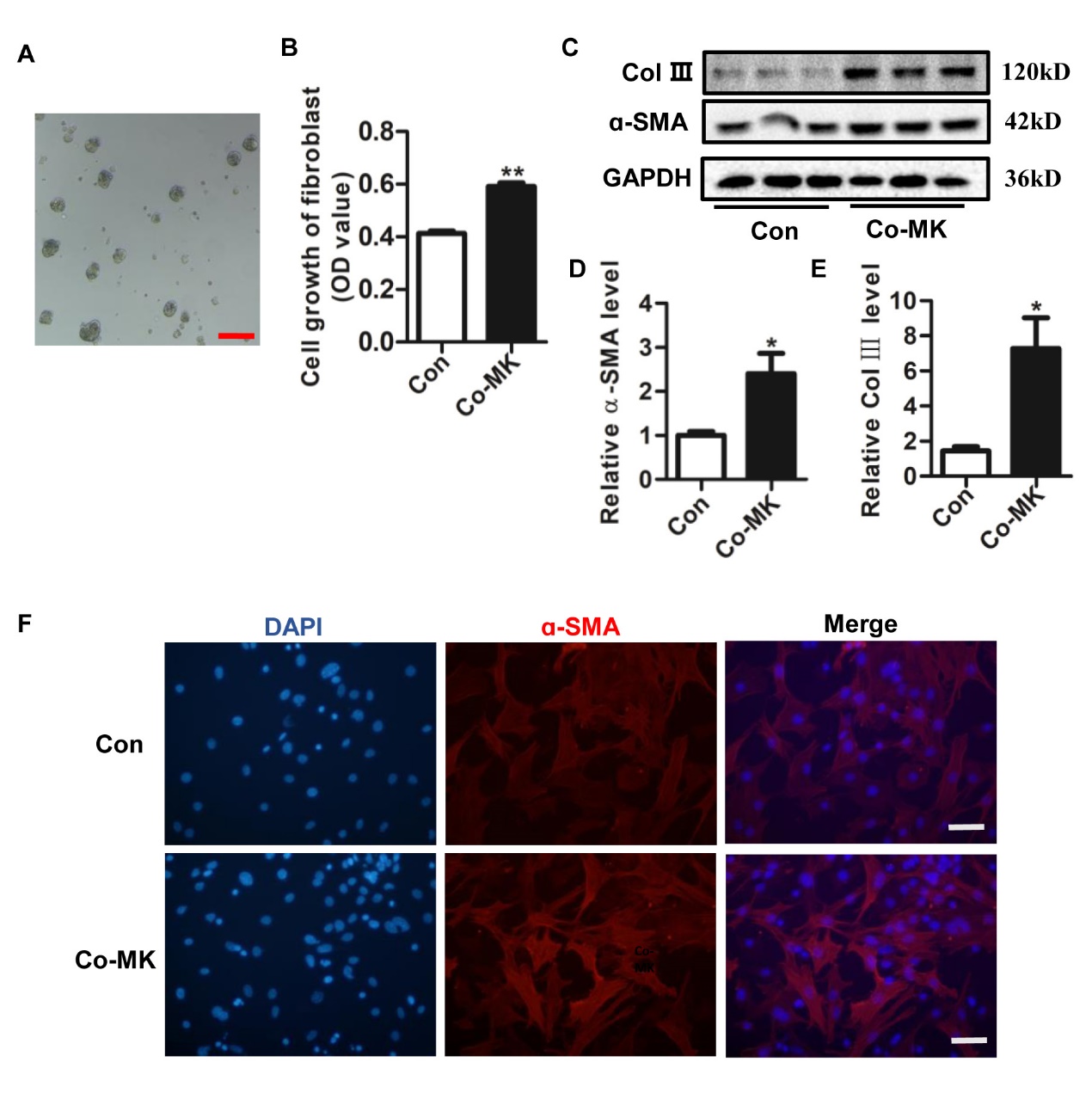
**

**Supplementary Figure S1** **Effects of Megakaryocytes from embryonic liver on the primary lung fibroblast.** (A) Microscopic appearance of primary megakaryocytes (Bar=50μm). (B) Cell growth of primary lung fibroblasts, evaluated by the CCK-8 assay (n=6, **P<0.01 vs control group). (C) The level of ɑ-SMA and Col Ⅲ evaluated by western blotting. (D) Semi-quantitative analysis of ɑ-SMA level evaluated by western blotting (n=3, *P<0.05 vs control group, Co-MK: fibroblast direct coculture with megakaryocytes). (E) Semi-quantitative analysis of Col Ⅲ level evaluated by western blotting (n=3, *P<0.05 vs control group, Co-MK: fibroblast direct coculture with megakaryocytes). (F) Immunofluorescence for ɑ-SMA of fibroblasts (Bar=60μm). The data are presented as the mean±SD.

**Supplementary Figure S1**


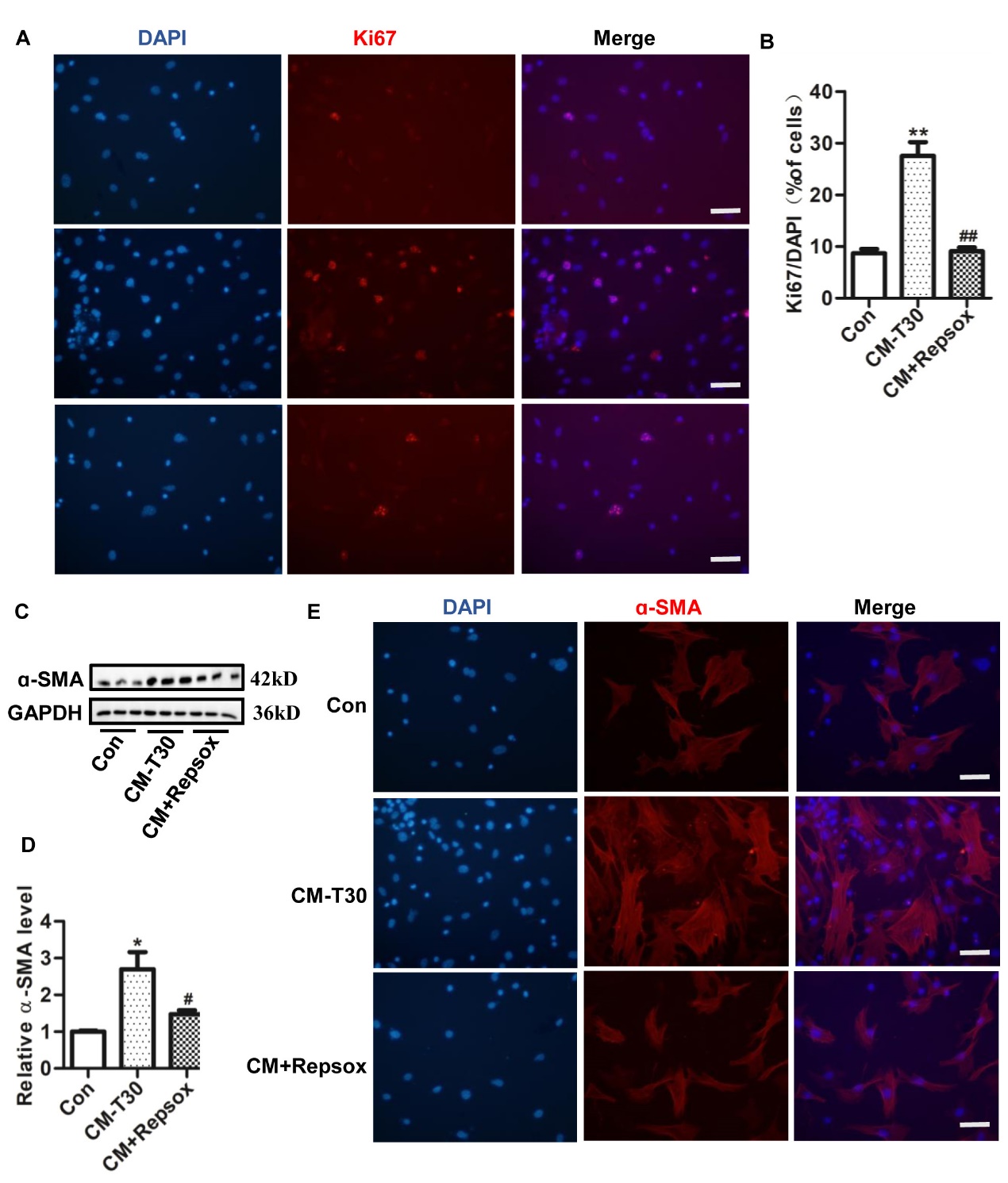


**Supplementary Figure S2 Effects of TPO-induced Megakaryocytes on the primary lung fibroblast.** (A) Immunofluorescence for Ki67 of fibroblasts (Bar=60μm). (B) Data analysis of the percentage of Ki67+ to DAPI (n=3, **P<0.01 vs control group. ##P<0.01 vs CM-T30 group). (C) The level of ɑ-SMA in fibroblast evaluated by western blotting. (D) Semi-quantitative analysis of ɑ-SMA level evaluated by western blotting (n=3, *P<0.05 vs control group. #P<0.05 vs CM-T30 group). (E) Immunofluorescence for ɑ-SMA of fibroblasts (Bar=60μm). The data are presented as the mean±SD.
